# Laboratory Evaluation of Mechanical Properties of Modified Asphalt and Mixture Using Graphene Platelets (GnPs)

**DOI:** 10.3390/ma14195599

**Published:** 2021-09-27

**Authors:** Mohamed Samir Eisa, Ahmed Mohamady, Mohamed E. Basiouny, Ayman Abdulhamid, Jong R. Kim

**Affiliations:** 1 Department of civil Engineering, Benha Faculty of Engineering, Benha University, Benha 13511, Egypt; mohamedeisa524@bhit.bu.edu.eg (M.S.E.); eng_ayman.salem@yahoo.com (M.E.B.); m.basiouny@yahoo.com (A.A.); 2 Department of Construction Engineering and Utilities, Faculty of Engineering, Zagazig University, Zagazig 44519, Egypt; dr_amohamady@yahoo.com; 3 Department of Civil and Environmental Engineering, School of Engineering and Digital Sciences, Nur-Sultan 010000, Kazakhstan

**Keywords:** graphene platelets, asphalt binder, hot mix asphalt, mechanical properties, laboratory evaluation

## Abstract

Recently, nanomaterials have attracted attention in the field of pavement construction as modifiers to endure heavy loads and climate changes. In this study, conventional asphalt (bitumen) of penetration grade AC (60/70) was modified with graphene platelets (GnPs) at three different contents: 0.5%, 1.0%, and 1.5% by weight of asphalt content. Kinematic viscosity, softening point, penetration, and dynamic shear rheology tests were performed to evaluate the mechanical properties of modified binder. The results showed that adding GnPs improves the mechanical properties of asphalt binder; the kinematic viscosities, softening points, and rutting parameters increased but penetrations decreased with the contents of GnPs. Hot mix asphalt specimens with GnPs-modified asphalt were prepared and characterized with Marshall tests, thermal stress restrained specimen tests (TSRST), wheel tracking tests, and indirect tensile tests. Similar to the results of asphalt binder, the mechanical properties of asphalt mixture were improved by GnPs. Marshall stability increased by 21% and flow decreased by 24% with accepted value of 2.8 mm in penetration when the mixture was modified with 1.0 wt% of GnPs. At the same GnPs content, modified asphalt mixture led to lower failure temperature by 2 °C in comparison with unmodified asphalt mixture and the cryogenic failure stress was improved by 12%. The wheel tracking tests showed that GnPs-modified asphalt mixture has outstanding deformation resistance in comparison with unmodified asphalt mixtures: after 5000 cycles, 1.0 wt% of GnPs reduced the rut depth of asphalt mixture by 60%—the rut depth of unmodified asphalt mixture was 6.9 mm compared to 2.75 mm for modified asphalt mixture. After 10,000 cycles, the modified asphalt mixture showed rut depth of 3.24 mm in comparison with 8.12 mm in case of unmodified asphalt mixture. Addition of GnPs into asphalt mixture significantly improved the indirect tensile strength: 1.0 wt% of GnPs increased the indirect tensile strength of unmodified asphalt mixture from 0.79 to 1.1 MPa recording ~40% increment. The results of this study can confirm that graphene platelets enhance the mechanical properties of asphalt mixture and its performance.

## 1. Introduction

The rapid growth and development of modern societies has led to complications in traditional transportation system. Heavy traffic loads accompanied with extreme weather conditions raise the urgent need to use robust asphalt mixture to pave roads in particular within the heavy traffic zones. High traffic loads urge the pavement industry to develop a robust pavement to stop early permanent deformation or cracks. Early pavement failure costs the city councils enormous amounts of money for maintenance, reconstruction, and traffic jams over the provided detours. Asphalt is an indispensable material in roadways, with high demand every year. It is a mix of aliphatic, aromatic, and naphthenic hydrocarbons and widely employed as protective and waterproof coatings, and binders in road construction. According to the Asphalt Institute [1], 87 million tons of asphalt are sold every year around the world; 85% of the production is employed in pavements.

The most common pavement failure is due to rutting, which is mainly due to non-homogenous dispersion of asphalt mixture and high temperature weather [2,3]. Fatigue cracks are also common in pavement due to aging phenomenon and/or low temperature weather. These failures can be prevented or delayed by modification and reinforcement of asphalt binder‒asphalt. Numerous modifiers were used to elevate the performance of asphalt mixtures including TAFPACK-Supper (TPS), crumbed rubber, styrene–butadiene styrene (SBS) copolymer, and a mixture of SBS and TPS [4,5,6,7,8,9,10]. Nanomaterials feature outstanding specific surface area and high aspect ratio leading to the development of high performance composites, and they have proved successfulness in enhancing mechanical performance and functional properties of construction and binding materials and polymers [11,12,13,14,15]. Recently, nanomaterials such as nanosilica [16,17], nanoclays [18], and nano-ZnO were employed to modify asphalt for hot asphalt mixture with long service life and high load capacity [4].

Organomodified nanoclays were extensively studied, resulting in high mechanical and thermal properties of asphalt [19,20,21,22,23,24]. Ashish’s group [20,22] modified asphalt grade AC-10 with nanoclay (CL-30B); rutting factor of asphalt was improved by 334% and performance index increased by 2-folds upon addition of 6 wt% of organomodified nanoclay (CL-30B). In another study, five asphalt nanocomposites were prepared and compared; asphalt/organoclay montmorillonite (OMMT), asphalt/nanoclay Cloisite^®^ 20Å (CLO), asphalt/styrene butadiene styrene (SBS), asphalt/OMMT/SBS, and asphalt/CLO/SBS nanocomposites [25]. Rheological and mechanical performances of modified asphalt were enhanced at different increments. El-Shafie et al. [26] showed that modified asphalt with nanoclay was less sensitive to high temperature changes; for example, the softening temperature increased by 13 °C, and plastic deformation was less in comparison with base asphalt. Polacco et al. [27] reported that the viscosity and stiffness of SBS-modified asphalt was increased when sodium montmorillonite (Na-MMT) and OMMT nanoclays added into asphalt leading to high rutting-resistance asphalt pavements [27]. 

Silica nanoparticles showed promising improvements when added into asphalt [28,29,30,31,32]. Karnati S. et al. [31] chemically modified silica nanoparticles (SnPs) by (3-aminopropyl) triethoxysilane (APTES) and mixed with asphalt. This led to a reduction in viscosity by 36% and 63% before and after aging, respectively. Pristine SnPs increased viscosity of asphalt by 100% (from 0.5 Pa.s to 1 Pa.s); after aging it further increased to 8 Pa.s. This was attributed to internal friction between SnPs and asphalt molecules. However, when SnPs were modified by APTES, the viscosity dropped by 36% and 63% before and after aging, respectively. The surface modification for SnPs worked as plasticizer or lubricant so that it diminished the friction between the particles and asphalt. Moreover, the study showed that surface modification for SnPs had a significant effect on viscosity aging index (VAI); the VAI of asphalt/ SnPs modified by APTES decreased by 50% in comparison with pristine SnPs [31]. Self-healing asphalt mixture was reported after simultaneously adding nanosilica and styrene butadiene styrene (SBS) polymer [15]; adding 2 wt% nanosilica and 4 wt% SBS into asphalt mixture promoted the rutting resistance and healing index due to the fast and effective filling of asphalt into microcracks.

Carbon-based nanomaterials such as carbon nanotubes and graphene exhibit striking mechanical and physical properties (1 TPa Young’s modulus, 130 GPa tensile strength, 2000–5000 W/m.K thermal conductivity, and 100–6000 S/cm electrical conductivity), and outstanding surface area and aspect ratio [33,34]. Therefore, scientists and researchers continuously explore their potential applications in different fields. Polymer/carbon-based nanomaterial composites have been extensively and steadily investigated, recording advanced progression in nanocomposites [33,35,36,37,38,39]. Recently, graphene and its derivatives have been recognized as promising additives to promote the mechanical performance of asphalt pavements and construction materials [40,41,42]. In a recent study, graphene oxide (GO) has promoted the rutting resistance of asphalt mixtures; 2 wt% of GO reduced the permanent deformation after 1000 cycles and permanent strain by 64.5% and 62.5%, respectively [40]. Zhu J. et al. [43] investigated the mechanical behavior of asphalt mixtures when GO was added. The results showed that GO not only enhanced the mechanical performance but also physical properties of asphalt mixtures; for example, adding 0.05 wt% GO, the penetration of the base asphalt (bitumen) was reduced by 32.5%; splitting tensile strength increased 41.4% when curing occurred at room temperature and incremented further 22.6% when the specimen was cured under freeze-thaw. Effect of graphene on softening point and penetration contradicts in the literature; for example, a study conducted by Li Y. et al. [44] showed that GO did not change softening point while penetration increased by 1% at 3 wt% GO; this increment can be due to experimental error. Kinematic viscosity and ductility are crucial properties to assess asphalt’s workability. Studies confirm that addition of stiff filler hardens the asphalt binder though it was within the accepted range. Ductility was decreased by 21% and kinematic viscosity (@75 °C) was significantly increased (by 900%) when 3 wt% GO was added to asphalt [44]. Despite the published studies on graphene-modified asphalt, the research on applying graphene in pavement industry is still far from real application, for two reasons: (i) methods that used to prepare GO are not scalable and the yield is in gram/sub-gram scale. (ii) GO is an undesirable graphene derivative and not environmentally friendly in the construction industry due to the large amounts of solvents and hazards used in its production process.

Graphene platelets (GnPs) are a derivative of graphene composed of few layers of graphene within range 1–5 layers [45]. GnPs have tensile strength of 101 GPa and Young’s modulus of 0.8–1 TPa [46]; these values are not far from those of single-layer graphene. The production cost of a defect-free, single-layer graphene is ~US$1000. In contrast, one kilogram of GnPs could cost about US$10–20 [46]. These GnPs were successful not only in reinforcing various polymers such as epoxy [47], PMMA [48], and elastomers [49] but also adding functionalities such as thermal and electrical conductivity and flame retardancy [50].

Taking into account the complexity, cost, and environmental concerns that are involved in synthesizing single-layer of graphene [45], with not much difference in mechanical properties, GnPs is considered as a promising candidate for low-cost and low-skill-required industry such as construction and polymer composites. In this study, a facile and scalable approach to fabricate few-layer graphene (1–5 layers) was used. This nanomaterial, named graphene platelets (GnPs), was fabricated by thermal shocking expandable graphite, followed by ultrasonication. The expandable graphite is not expensive, and its production is well established. Conventional asphalt (bitumen) of penetration grade AC (60/70) was modified with graphene platelets (GnPs) and the mechanical properties of modified asphalt binder and its mixture were investigated in the laboratory. 

## 2. Materials and Methods

Graphite intercalated compounds (Asbury 3494, GICs) were provided from Asbury Carbons, Asbury, NJ, USA. GIC contains carbon content of 80% and size of 75 μm. Asphalt with penetration grade 60/70 was purchased from Suez Refinery Company, Suez, Egypt. All other materials used in this work include aggregate, sand, and mineral filler were obtained from the Egyptian Transportation Organization and used as received without further purifications. 

Aggregate generally accounts for 92 to 95 percent of asphalt concrete mixtures. Crushed lime-stone aggregates and sand were collected from Suez Quarry, Suez, Egypt and used in this study. They were divided into two types: coarse and fine aggregates. Coarse aggregates are portions that are retained on a sieve of size 2.36 mm while fine aggregates are retained on range 0.075–2.36 mm. The properties of the aggregates are presented in Table 1.

Asphalt is a high viscosity binder which is extracted by crude oil distillation process under high pressure and temperatures. Asphalt exists in wide range of properties; they differ in melting and freezing temperatures, flowability, and viscosity. Herein, asphalt of penetration grade of 60/70 was employed, and summary of its properties is presented in Table 2. Asphalt was purchased from Suez Petroleum Refining Company, Suez, Egypt.

### 2.1. Material Preparation 

#### 2.1.1. Graphene Platelets

**Figure 1:**
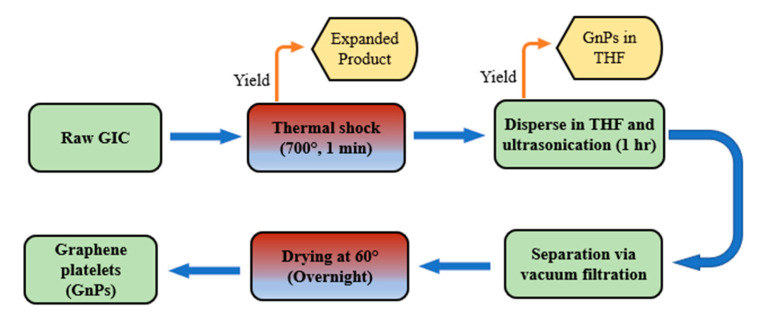
Preparation chart for graphene platelets to be ready for mixing with asphalt.

In this study, a simple approach was used to fabricate graphene platelets which included a thermal shock and ultrasonication. First, one gram of GICs (expandable graphite) was thermally treated at 700 °C in a preheated crucible for one min in a commercial furnace (DF202, 4000 W, Deng Yang Instruments Co., Ltd., Beijing, China). Upon thermal shocking, the graphite intercalation compounds expanded into a worm-like and fluffy structure. A desired weight of the expanded product was dispersed in tetrahydrofuran (THF) by mechanical stirring for 10 min, followed by sonication for 1 h in a metallic container. The sonication facility has a power capacity of 200 W and frequency of 42 kHz; thereby, the ultrasonic waves surpassed the weak van der Waal forces between the GIC stages and graphene layers. The ultrasonic waves delaminated the fluffy structure into mono and few-layer graphene (graphene platelets). The produced graphene platelets (GnPs) washed through filter-cone device and then dried in a ventilated oven at 120 °C overnight to remove any traces of the solvent. Figure 1 illustrates a brief description of the fabrication method. 

#### 2.1.2. Nanocomposites Preparation 

**Figure 2:**
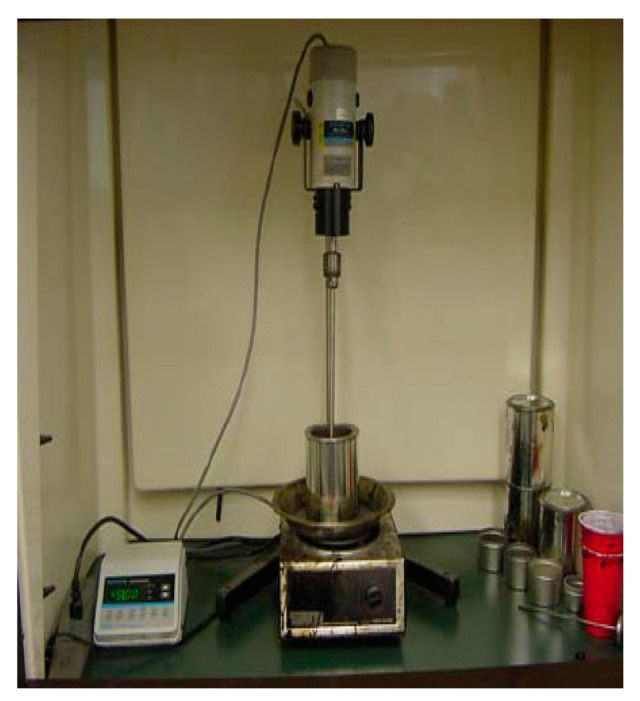
Mechanical mixer for stirring base asphalt with graphene platelets.

Asphalt is the base asphalt binder for all the prepared hot mix asphalt. Asphalt/GnPs nanocomposites were prepared as follows. Asphalt was liquified by heating up to 150 °C [51]. Then, a predetermined weight of GnPs was added and mixed with liquid-asphalt using high-speed mechanical mixer (Figure 2); mixing speed was set at 3000 rpm and temperature was kept at 150 °C using hot plate. The mixing operation took place for 1 hr to obtain homogeneous dispersion. Three weight fractions of asphalt/GnPs nanocomposites were prepared: 0.5, 1, and 1.5 wt%. Unmodified asphalt was liquified and processed following similar procedures to obtain a control sample. Asphalt mixture including asphalt/GnPs nanocomposite and aggregates were mixed together at high temperature, 150 °C. The low viscous mixture was then poured into a preheated mold and packed using Marshall hammer of 4.5 kg weight. Finally, the compacted samples were left overnight to cool down to room temperature. 

### 2.2. Asphalt Mixture Design

**Figure 3:**
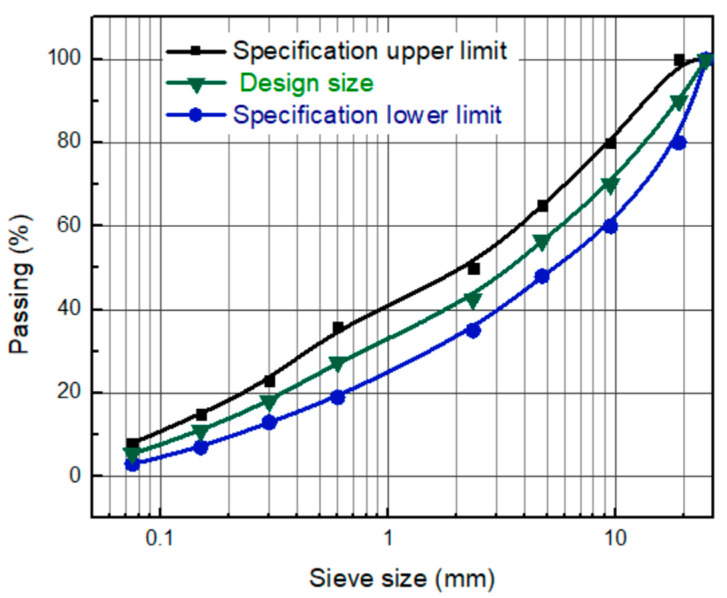
Aggregate gradation of the mix design.

In this study, technical specifications of Egyptian Transportation Organization for Highway and Bridges Construction were followed. Crushed lime-stone aggregates, sand, and filler were graded in laboratory sieves to fall within the upper and lower limits, according to the Egyptian Code of Practice for urban and rural roads (ECP, 2008) [52]. Figure 3 presents the aggregate gradation used in the current study for wearing surface layer (heavy traffic volume). Marshall method [53] was employed to determine the optimum percentage of asphalt in the asphalt mixture and accordingly asphalt percentage was set at 5.2 wt%. All prepared asphalt mixtures comprised of same gradation and optimum asphalt content. 

### 2.3. Test Methods

#### 2.3.1. Measurements of GnPs and Their Asphalt Nanocomposites 

##### Morphology of Graphene Platelets (GnPs) 

Morphology of GnPs was observed using scanning and transmission electron microscopies. Scanning electron microscopy was employed to study the morphology of the precursor (graphite intercalated compounds) and expanded product after thermal shocking. The sample was coated by thin layer of platinum and observed by (SEM, Philips XL30 Feg,) at 5 kV. Transmission electron microscopy (H-800-1 TEM, Hitachi Co., Tokyo, Japan) was used to study the nano-morphology of the developed graphene platelets. The scanning was carried out at 200 kV. TEM sample was prepared as following: GnPs were suspended in THF solvent by sonication; the suspension underwent a sequence of diluting process to reach 0.0001 wt% by sonication. A few drops were dropped on a 200-mesh copper grid and dried in a ventilated oven at 120 °C. 

##### Physical Properties of Asphalt/GnPs Nanocomposites

Properties of unmodified asphalt and its GnPs-nanocomposites were measured including softening point, kinematic viscosity, and penetration test. Softening point was measured using ball-and-ring apparatus at temperature range 30–157 °C according to standard ASTM-D36/D36M [54]. Kinematic viscosity of unmodified asphalt and asphalt/GnPs nanocomposites was determined using DV2T viscometer, spindle type (AMETEK Brookfield Co.). The kinematic viscosity measurements were conducted at 100, 120, 135, 150, 160, and 170 °C according to standard ASTM-D4402 [55]. A temperature-controlled thermal chamber was used to maintain the sample temperature constant at the pre-set value.

**Figure 4:**
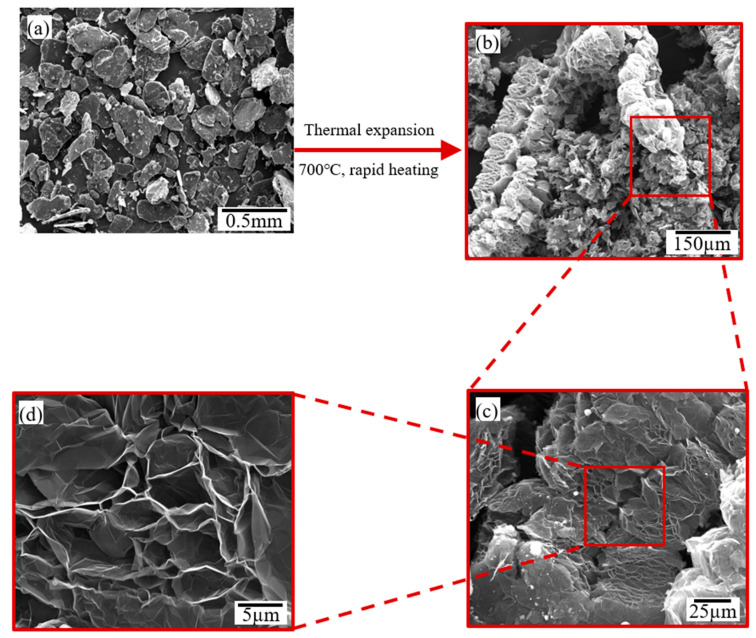
SEM images of (**a**) graphite intercalated compounds (GICs) and (**b**–**d**) low to high magnification images for expanded GICs after thermal shock at 700 °C for one min.

Penetration test (@25 °C, 0.1 mm) was conducted on unmodified asphalt and its GnPs-nanocomposites following standard AASHTO T49 [56]. The pull speed and loading time in the penetration test were 50 mm/min and 5 sec, respectively. Figure 4 shows digital images of the instruments used to measure physical properties of GnPs-based asphalt composites.

##### Complex Modulus and Phase Angle 

The dynamic shear rheology (DSR) was used to determine complex modulus (G*) and phase angle (δ) according to AASHTO T 315 [57]; both parameters are indicators that determine stiffness of asphalt at high temperature (rutting resistance). Auto-rheometer (type CVO100) manufactured by Bohlin Instrument was employed to conduct DSR measurement. A thin film of asphalt was sandwiched between two circular plates. The fixed plate is the lower while the upper plate oscillates forth and back across the sample to create shearing action at frequency of 10 rad/sec; this level of frequency simulates shearing action created by traffic at speed of 90 km/hr according to Superpave specifications [58]. 

DSR was applied on unaged and short-term aged samples. Laboratory aging was carried out through rolling thin-film oven test (RTFOT) procedures according to standard -ASTM D1754 [59]. The test was conducted on unmodified asphalt binder and GnPs-modified asphalt at 0.5 wt%, 1.0 wt%, and 1.5 wt% at temperatures 58, 64, 70, and 76 °C, respectively. DSR sample has dimensions of 1 mm thickness and 25 mm in diameter. Complex shear modulus (G*) and phase angle (*δ*) were recorded during the DSR measurement. Rutting parameter (*RP*) was calculated using Equation (1):

(1) $$RP=\frac{G^{*}}{\sin\delta }$$

#### 2.3.2. Measurements of Asphalt Concretes

##### Marshall Test

Marshall test was conducted on asphalt mixtures to measure their stability and resistance to plastic deformation. The test was carried out according to ASTM D6927 [60] in which a compacted and cylindrical sample with GnPs content range 0–1.5 wt%. The specimen was 100 mm in diameter and 63.5 mm in height. As per standard, each specimen was soaked into water at 60 °C for 30 min. Then the sample was loaded in compression mode at constant loading speed of 5 mm/min. Marshall stability is measured as the maximum load attained by the sample at fracture. The total deformation at the maximum load (at failure) was recorded to define the flow value. Three samples of each fraction were tested to obtain the average. 

##### Wheel Tracking Test

Wheel tracking was used to assess the resistance of asphalt mixtures against rutting at high temperatures and under dynamic loads. Wheel-tracking test was conducted on an asphalt concrete slab with dimensions of 265 mm × 320 mm × 40 mm according to standard AASHTO T-324 [61]. The sample was first heated to 60 °C and kept at this temperature for 6 hrs. Then, the test was carried out at 60 °C under wheel load of 70 kg. The test ran up to 10,000 cycles with a rut-gauge of 0.001 mm precision. 

##### Thermal Stress Restrained Specimen Test 

The thermal stress restrained specimen test (TSRST) was carried out to evaluate the thermal cracking resistance of asphalt concrete mixtures according to EN 12697-46 standard [62]. Low temperature cracking is attributed to tensile stresses induced in asphalt concrete pavement when the temperature drops to a severe low temperature, which developed in the pavement as a result of the pavement’s contraction.

A test specimen (35 mm × 35 mm × 210 mm) was epoxied to two aluminum plates and left for curing. Then, the specimen and plate assembly were mounted into the load frame. TSRST was conducted by cooling the asphalt specimen at a constant rate of 10 °C/h while maintaining the specimen at constant length. The contraction of the specimen during the cooling process was measured using two linear variable differential transducers. The test was run up to sample fracture where the stress exceeded the cryogenic strength. The parameters obtained from TSRST were fracture temperature and fracture strength.

##### Indirect Tensile Strength Test

The indirect tensile strength test is a crucial test to indicate crack resistance of asphalt concrete. The tensile stresses resistance of asphalt mixture was conducted using the Marshall specimen where the sample was subjected to a compressive load acting along a diametric plane at room temperature and constant rate of 51 mm/min at room temperature. The specimen diameter was 101 mm and the width was 13 mm as per standard ASTM D 6931 [63]. Plywood loading strips were used to maintain a uniform distribution of the load along the length of specimen. The tensile load in the horizontal direction of the specimen is indirectly generated by the compressive load. The peak value of the load at failure was recorded to determine the tensile strength by using Equation (2):

(2) $$ITS=\frac{2\;P_{max}}{\pi \;t\;D}$$

where *ITS* is indirect tensile strength in MPa; *P_max_* is the maximum load at failure in N; and *t* and *D* are specimen height and specimen diameter in mm before the test, respectively.

## 3. Results and Discussion

### 3.1. Morphology of Graphene Platelets

Scanning electron microscopy (SEM) analysis was performed to visualize synthesis stages of graphene platelets. Figure 4 displays micrographs of graphite intercalated compounds and worm like structure of the expanded product at different magnifications. Image (a) confirms the flake structure of the intercalated graphite; plates of intercalated graphite are obviously shown with thickness and lateral dimensions in micro scales. Image (b) depicts the worm-like structure of the expanded product. When the intercalated graphite is thermally shocked at high temperature, the intercalants, between graphite layers, evaporate, producing enormous pressure leading to high volume expansion and producing the worm-like structure presented in Image (b). When a randomly selected portion is magnified in image (c), the expanded product shows a loose and fluffy carbon product which facilitates the exfoliation process later upon ultrasonication. Further magnification to Image (d) shows thin sheets with corrugated surface due to thermal shock.

**Figure 5:**
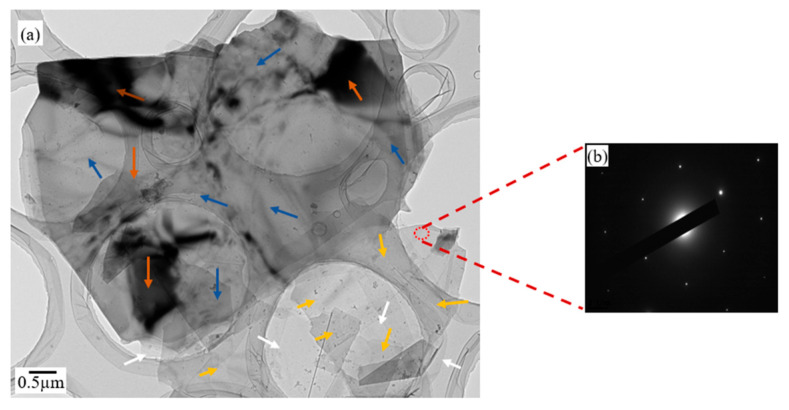
(**a**) TEM image of graphene platelets after one-hour ultrasonication and (**b**) electron diffraction pattern of a randomly selected area.

Transmission electron microscopy (TEM) was used to observe the nanostructure of the graphene platelets after one-hour ultrasonication. Figure 5a displays the image of graphene platelets sitting on lacey-carbon support. It evidently shows plat-like structure of the developed graphene platelets. The progression in color intensity demonstrates the corresponding number of graphene layers: featureless and transparent areas (indicated by white arrows) represent single graphene layer; high coloring intensity areas depicted by blue arrows illustrate multi-layer graphene (~5 layers [39]); average intensity areas demonstrated by yellow arrows contain two to three graphene layers. Dark areas denoted by brownish arrows signify many layers of graphene piled up. The progression in number of layers was expected due to overlapping between layers, yet they are likely to separate into the matrix provided that appropriate mixing approach is employed. The developed GnPs possessed thickness within range ~2–5 nm. These results were similar to the literature [33,53,54]. Crystalline structure of GnPs was examined by studying the electron diffraction pattern of a featureless and transparent where a single layer of graphene is expected. Figure 5b shows a typical symmetric six folds confirming highly crystalline structure of GnPs. This procedure is in accordance with the literature [39]. 

### 3.2. Workability of Asphalt and Its GnPs-Modified Composites

**Figure 6:**
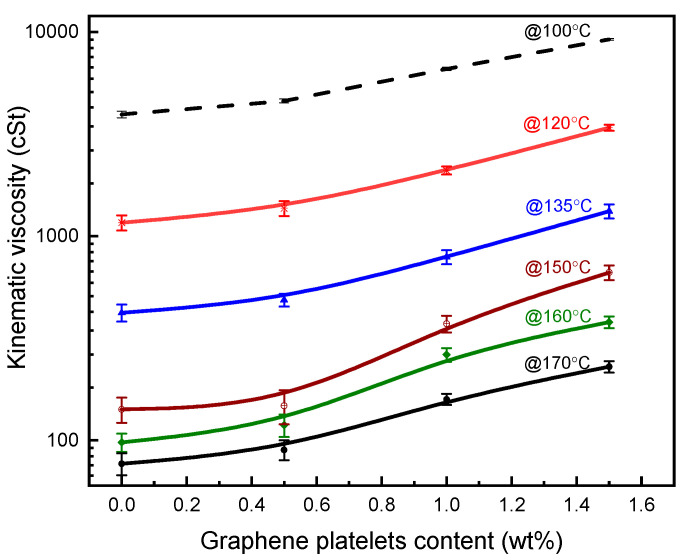
Kinematic viscosity of unmodified asphalt and its graphene platelets nanocomposites within temperature range 100–170 °C.

In this study, kinematic viscosity, softening point, and penetration measurements were conducted to determine the influence of graphene platelets on the physical properties and workability of asphalt 60/70 grade. Viscosity is a measure of resistance for a fluid to flow due to shear or tensile stresses; it is a fundamental parameter for rheological properties of asphalt. Investigating the relationship between viscosity and temperature helps to identify the workability range to achieve a coherent, cohesive, and homogeneous asphalt mixture; high viscosity will lead to incomplete coating for the asphalt aggregate by the asphalt whereas low viscosity means asphalt drainage is likely to happen [64]. Figure 6 displays kinematic viscosity of unmodified asphalt and its GnPs-modified asphalt with temperature range of 100–170 °C and graphene content (0–1.5 wt%). Kinematic viscosity of all samples dropped with the increase of temperature; for example, unmodified asphalt had a viscosity of 422 cSt at 135 °C and it reduced to 142 cSt at 150 °C. A similar trend was observed for GnPs-modified asphalt samples.

Adding GnPs into asphalt increased its kinematic viscosity at all fractions; 1.5 wt% of GnPs increased the viscosity of asphalt from 422 to 1327 cSt, recording 214% increment at 135 °C. At high temperature, GnPs posed significant effect on the viscosity of asphalt in comparison with less effect at low temperature. For example, at 120 °C, the kinematic viscosity of asphalt was 1370 cSt at 0.5 wt% GnPs and increased to 3400 cSt at 1.5 wt% GnPs, recording an increment of 148%. At 160 °C, asphalt viscosity was 119 cSt at 0.5 wt% of GnPs and increased to 380 cSt at 1.5 wt%, recording 219% increase. 

This could be explained as follows. In general, adding graphene platelets into asphalt creates friction between the platelet surface and asphalt molecules which in turn increases the latter’s flow resistance, i.e., viscosity. The viscous friction occurs due to three motions (i) relative motion between long chain molecules of asphalt, (ii) relative motion between graphene surface and asphalt molecules, and (iii) relative motion between graphene-graphene surfaces. Therefore, more graphene platelets will generate more friction which added to the high inherit asphalt flow resistance; this ultimately increases the flow resistance at high GnPs loadings. High viscosity GnPs-modified asphalt resists deformation at high temperature and, consequently, resists rutting and heavy traffic loads.

**Figure 7:**
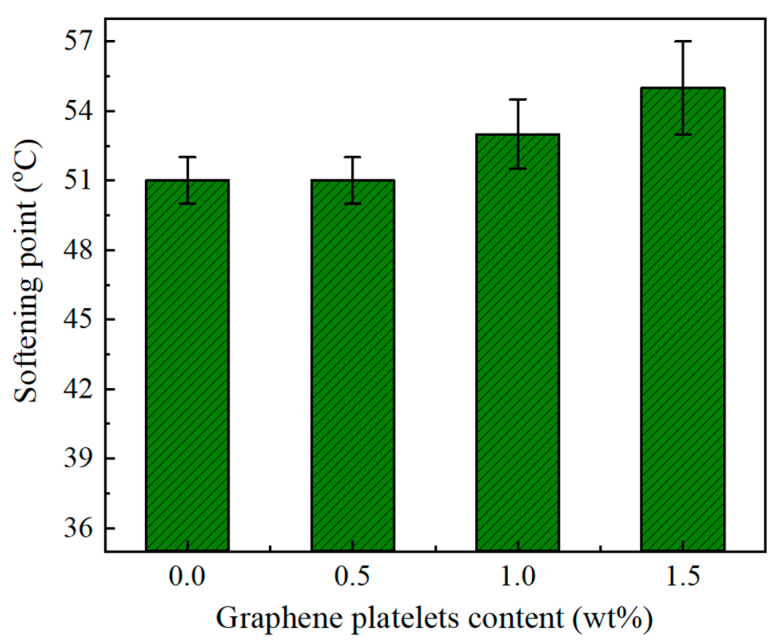
Softening point of unmodified asphalt and its graphene platelets nanocomposites.

**Figure 8:**
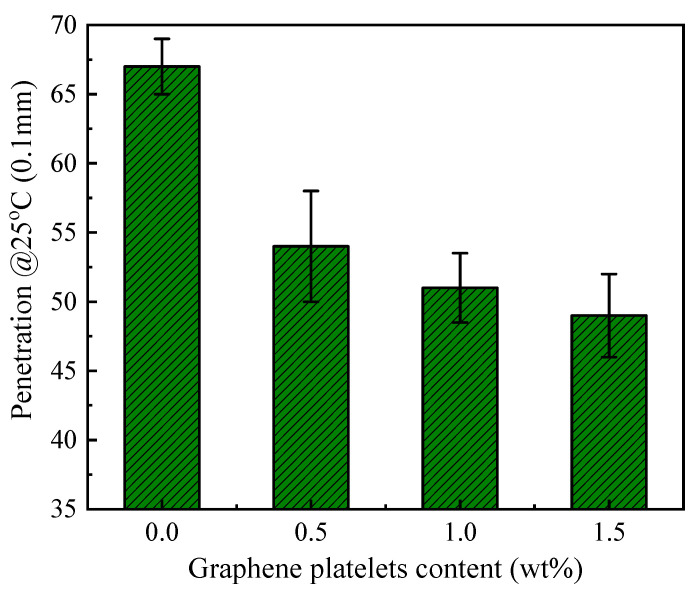
Penetration level of unmodified asphalt and its graphene platelets nanocomposites at 25 °C @0.1 mm.

Figure 7 contains the values of softening point of unmodified asphalt and GnPs-modified asphalt at various GnPs contents. The measurements showed that GnPs increased the softening point of asphalt; GnPs-modified asphalt possessed softening point of 55 °C at 1.5 wt% of GnPs in comparison with 51 °C for unmodified asphalt. This is in favor of the asphalt’s performance in asphalt mixture at high temperature, i.e., the hot asphalt mixture would be stiffer with GnPs-modified asphalt and can resist deformation. These results are in accordance with previous results where graphene oxide was used as an additive to modify asphalt for high rheological performance [65]. Penetration (@25 °C, 0.1 mm) is a common test for asphalt samples. It measures the relative stiffness of the prepared bituminous samples; low penetration means high-stiffness asphalt. In penetration test, at least three measurements of each sample were performed the average was recorded. Figure 8 shows penetration test results of unmodified asphalt (60/70 grade) and its GnPs-nanocomposites. It is evident that adding GnPs into asphalt enhances its resistance to deformation; penetrations reduced with the GnPs content. For example, at 1.5 wt% of GnPs, the penetration depth reduced by 27% in comparison with unmodified asphalt. This indicates that asphalt/GnPs nanocomposites became stiffer with the addition of graphene filler. This is in agreement with the measurements of viscosity where asphalt had higher resistance to flow and hence high resistance to permanent deformation by adding GnPs. The significant decrease of asphalt penetration reduces the chance of rutting process and enhances the high-temperature performance of asphalt mixture. This result is important for the roads and pavement in the hot climate regions. 

### 3.3. Rheological Properties of Asphalt and Its GnPs-Based Composites

**Figure 9:**
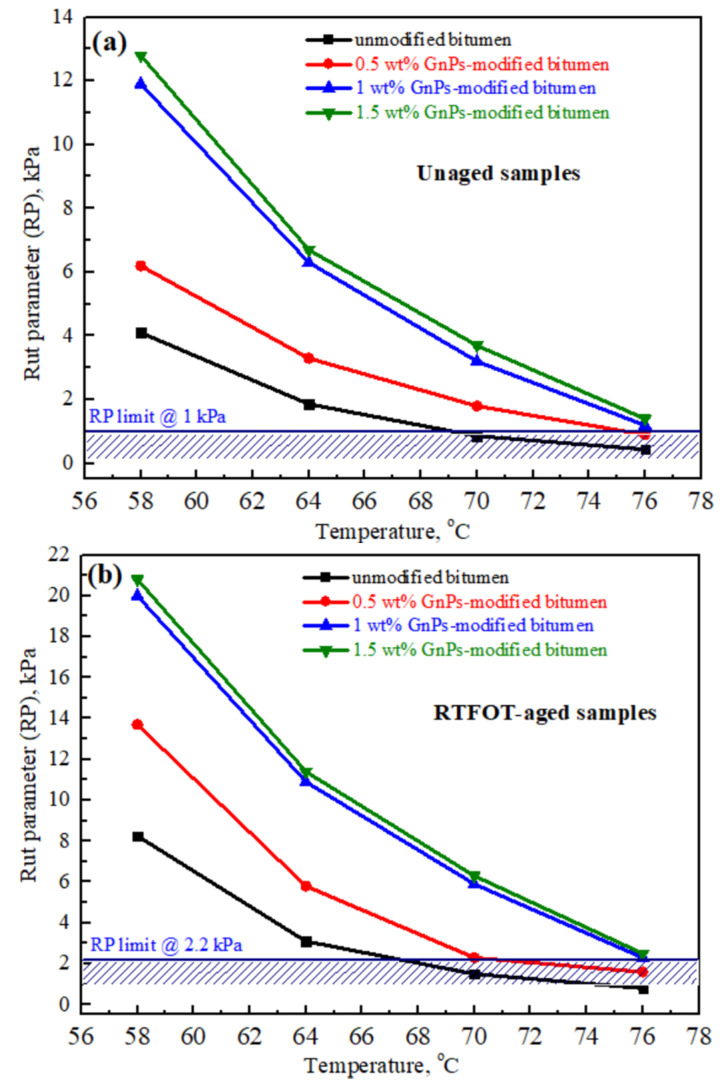
Rut parameter of unmodified asphalt and its GnPs nanocomposites after conditions of (**a**) unaging and (**b**) RTFO-aging.

Figure 9a and b show rutting parameter of unaged and RTFOT-aged samples of unmodified asphalt and their GnPs composites as a function of temperature and wt% of GnPs. According to standard [66], the rutting parameter limits were set—within the selected temperature—to:

(i) RP ≥ 1 kPa for unaged asphalt
(ii) RP ≥ 2.2 kPa for RTFOT-aged asphalt

Rutting parameter of unaged asphalt is shown in Figure 9a; it increased with the content of GnPs while it steadily decreased with the increase in temperature. For example, at 1.5 wt% GnPs, the rutting parameter increased from 4 kPa for unmodified asphalt to 12.9 kPa, recording 3.2-fold increment at 58 °C. High temperature decreased the rutting parameters below the boundary (1 kPa) for unmodified asphalt with a threshold temperature of 70 °C. On the other hand, modifying the asphalt with GnPs increased its viscosity. Thus, it can withstand shear stresses and acquire high rutting resistance at high temperature; the threshold temperature for all GnPs-modified asphalt was ~76 °C. Additionally, it was noted that the rutting parameter of 1.5 wt%-GnPs-modified asphalt was higher than that of 1.0 wt%-GnPs-modified asphalt at any temperature but the increment was not significant. 

Figure 9b shows the rutting parameter of RTFOT-aged samples. It is depicted that asphalt became stiffer after aging: the rutting parameter of unmodified asphalt after aging increased to 8 kPa in comparison with 4 kPa for unaged sample at 58 °C. This is a crucial advantage of GnPs-modified asphalt in comparison with the unmodified asphalt in prolonging service life of asphalt concrete with less maintenance costs. Similar to the case of the unaged asphalt, the rutting parameter of 1.5 wt%-GnPs-modified asphalt was higher than that of 1.0 wt%-GnPs-modified asphalt at any temperature but the increment was not significant. 

The threshold temperatures were dropped by ~3 °C after aging for both unmodified and 0.5 wt%-GnPs-modified asphalt (from 70 °C to 67 °C for the unmodified asphalt and 76 °C to 72 °C for the 0.5 wt%-GnPs-modified asphalt.) However, high contents of GnPs (1 wt%) kept the threshold temperature unchanged after aging (76 °C). 

### 3.4. Marshall Stability and Flow of Asphalt Mixture

Marshall mixing method is a widely used approach to determine the binder (asphalt) quantity in hot mix asphalt mixtures. The method is based on two main factors (i) volumetric analysis in terms of density and voids in the prepared samples and (ii) the stability and flow of asphalt mixture [67]. Since GnPs were added at low fractions, the binder contents in unmodified asphalt mixture were the same for all GnPs-modified asphalt samples.

**Figure 10:**
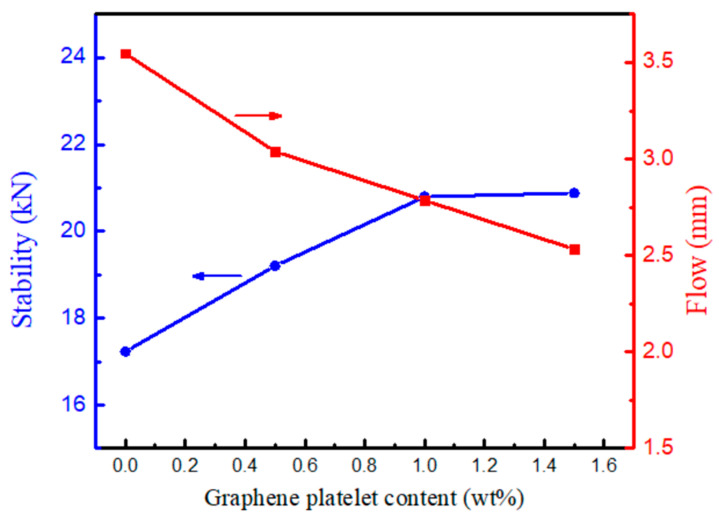
Marshall stability and flow resistance depth of asphalt mixes containing GnPs.

Figure 10 shows Marshall stability and flow as function of GnPs content. It demonstrates that asphalt mixtures possessed higher stability in comparison with unmodified asphalt mixtures. The maximum Marshall stability was observed at 1 wt% GnPs recording 2120 Kg compared to 1756 Kg for unmodified asphalt mixture—21% increment; as depicted in Figure 10; the stability did not increase much after 1.0 wt% of GnPs. Additionally, it was noticed that asphalt flow was reduced upon adding GnPs: 1.0 wt% of GnPs reduced the flow of asphalt mixture from 3.54 mm in unmodified asphalt mixture to 2.7 mm (24% reduction), which is still within the Marshall flow limit for all traffic levels −2 mm [67]. It confirms that GnPs increase the deformation resistance of asphalt mixture without susceptibility to premature cracking. The large specific surface area along with the high stiffness inherited in GnPs are the main reasons for achieving high Marshall stability; GnPs were able to create interface with asphalt and thus form physical barrier to allow binder and aggregates to flow resulting in asphalt mixtures with high strength and stability.

Based on the results of Marshall stability tests (the stability did not increase much after 1.0 wt% of GnPs and its flow is within the Marshall flow limit for all traffic levels—2 mm), only 1.0 wt% GnPs modified asphalt mixture with 1.0 wt% GnPs was used for the further study.

### 3.5. Low Temperature Cracking Resistance 

**Figure 11:**
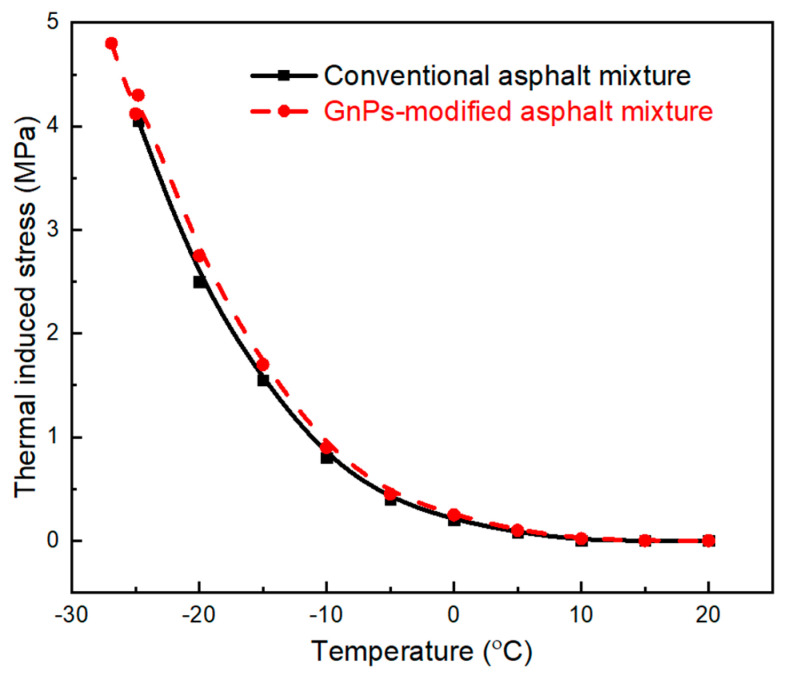
Thermal induced stress of unmodified asphalt mixtures and GnPs-modified asphalt mixture.

Low temperature cracking resistance of unmodified asphalt mixture and the GnPs-modified one was investigated via performing a thermal stress restrained specimen test. Figure 11 presents the thermal stress restrained specimen test (TSRST) results for the asphalt mixtures. The test started at temperature *T_o_* = +20 °C while the cooling rate was set at 10 °C/h. Table 3 contains recorded values of failure temperature and cryogenic stress of unmodified and GnPs-modified asphalt mixtures. Adding 1.0 wt% GnPs into asphalt led to lower failure temperature by 2 °C in comparison with unmodified asphalt mixture; modified asphalt showed −24.8 °C while the unmodified asphalt recorded −26.9 °C. Additionally, stresses of GnPs-modified asphalt displayed higher values than that of unmodified asphalt mixture; (i) cryogenic failure stress was improved by 12% for GnPs-modified asphalt mixture; and (ii) cryogenic stress at −20 °C was increased from 2.5 MPa for unmodified asphalt mixture to 2.75 MPa in case of GnPs-modified asphalt mixture. This is attributed to the outstanding mechanical properties of graphene (stiffest and strongest material ever measured) which improved the tensile strength of asphalt mixture, thus increasing low temperature cracking resistance. The results are consistent with the physical properties of asphalt/GnPs nanocomposites in the Section 3.2.

### 3.6. Deformation Performance

**Figure 12:**
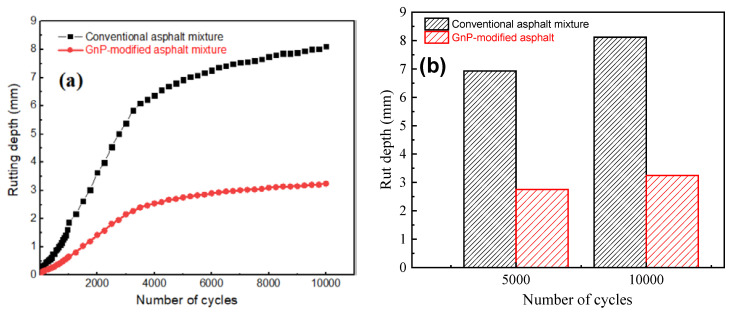
Rutting depth analysis of unmodified asphalt mixture and 1 wt% GnPs-modified asphalt mixture.

The accumulative irreversible deformation in pavement materials and asphalt mixture under cyclic loading is crucial in road pavement design. Rutting failure in pavement is mainly due to severe plastic and viscoelastic (or permanent) deformation [2,3]. Various tests are used to examine the deformation performance of asphalt mixtures including wheel tracking test, constant strain and stress rate tests, and direct stiffness measurements. In the current work, wheel tracking test at 60 °C and 70 kg load according to standards AASHTO T-324 [61] under dry conditions. Wheel tracking test presents real-load scenario at high temperature and humidity. The test was conducted on two samples: unmodified asphalt mixture and 1.0 wt% GnPs-modified asphalt mixture. Figure 12a displays measurements of rut-depth versus number of sweeping passes (cycles). Obviously, GnPs-modified asphalt mixture has outstanding deformation resistance in comparison with unmodified asphalt mixtures: in Figure 12b, after 5000 cycles, 1.0 wt% of GnPs reduced the rut depth of asphalt mixture by 60%—the rut depth of unmodified asphalt mixture was 6.9 mm compared to 2.75 mm for 1 wt% GnPs-modified asphalt mixture. For the first 5000 cycle, rutting is 6.9 mm, which means about 85% of rutting happened in the first 5000 cycle and 15% of rutting happened in subsequent 5000 cycles. A significant contrast in rut depth of unmodified and GnPs-modified asphalt mixtures was recorded after 10,000 cycles: the modified asphalt showed rut depth of 3.24 mm in comparison with 8.12 mm in case of unmodified asphalt mixture. 

Permanent deformation resistance of asphalt was significantly enhanced upon incorporating GnPs due to the following reasons: (i) graphene possesses excellent stiffness and strength and thus reinforces asphalt mixture to oppose plastic deformation; (ii) the plate-like structure and large specific surface area of graphene platelets increase interfacial strength between GnPs and asphalt; this would allow GnPs to effectively contribute to the deformation resistance; and (iii) large surface area of GnPs increases the viscosity of asphalt mixture and hence hinder layer slippage at high temperature in comparison to unmodified asphalt mixture.

### 3.7. Indirect Tensile Strength Test 

**Figure 13:**
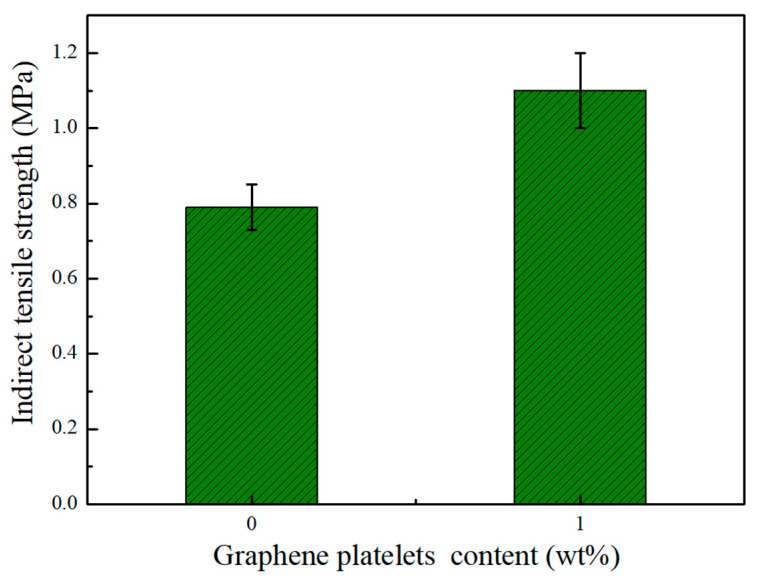
Indirect tensile test results of unmodified asphalt mixtures and GnPs-modified asphalt mixture.

There are different methods to measure the strength of asphalt mixture that can confirm its ability to withstand heavy vehicle loads without significant permanent deformation. Slow-crack propagation within the asphalt mixture is one of the objectives of modifying asphalt. Indirect tensile strength (*ITS*) is an important measure to evaluate crack resistance of asphalt concrete. In this work, two asphalt samples were prepared: unmodified asphalt mixture (0 wt% GnPs) and GnPs-modified asphalt mixture (1.0 wt%). Each sample was replicated three times to obtain the average. Figure 13 displays the results of *ITS* test for both unmodified and GnPs-modified asphalt mixtures. Addition of GnPs into asphalt mixture significantly improved the ITS; 1.0 wt% of GnPs, increased the ITS of unmodified asphalt mixture from 0.79 to 1.1 MPa recording ~40% increment. This improvement in tensile strength is a key indicator for high pavement resistance to cracks and longer life span in comparison with unmodified asphalt mixture.

A major part of tensile stress generated in asphalt mixture is endured by asphalt which binds aggregates together. When GnPs is added into asphalt, it enhances the binding between modified asphalt and aggregates. Additionally, GnPs can bridge initiated microcracks and work as crack hinders, slowing their propagation. This could be the reason for having high tensile strength of asphalt mixture upon the addition of GnPs. 

## 4. Conclusions

In this study, the mechanical properties of modified asphalt binder and its mixture were evaluated in the laboratory. The conventional asphalt (bitumen) of penetration grade AC (60/70) was modified with graphene platelets (GnPs) at three different contents: 0.5%, 1.0%, and 1.5% by weight of asphalt content. 

Scanning electron microscopy (SEM) analysis and transmission electron microscopy (TEM) were used to observe the morphology of GnPs. After one-hour ultrasonication, the synthesized graphene platelets (GnPs) comprised of 2–5 layers and the thickness of GnPs was in range 3–5 nm with high structural integrity. 

Kinematic viscosity, softening point, penetration, and dynamic shear rheology tests were performed to evaluate the mechanical properties of modified binder. The results showed that adding GnPs improves the mechanical properties of asphalt binder; the kinematic viscosities, softening points, and rutting parameters increased but penetrations decreased with the contents of GnPs. 

For the mechanical evaluation of asphalt mixture, Marshall tests, thermal stress restrained specimen tests (TSRST), wheel tracking tests, and indirect tensile tests were employed. Similar to the results of asphalt binder, the mechanical properties of asphalt mixture were improved by GnPs. Marshall Stability improved by 21% and flow reduced by 24% with accepted value of 2.8 mm in penetration when the mixture was modified with 1.0 wt% of GnPs. At the same GnPs content, modified asphalt mixture led to lower failure temperature by 2 °C in comparison with unmodified asphalt mixture and the cryogenic failure stress was improved by 12%. The wheel tracking tests showed that GnPs-modified asphalt mixture has outstanding deformation resistance in comparison with unmodified asphalt mixtures: after 5000 cycles, 1.0 wt% of GnPs reduced the rut depth of asphalt mixture by 60%—the rut depth of unmodified asphalt mixture was 6.9 mm compared to 2.75 mm for 1.0 wt% GnPs-modified asphalt mixture. After 10,000 cycles, the modified asphalt mixture showed rut depth of 3.24 mm in comparison with 8.12 mm in case of unmodified asphalt mixture. Addition of GnPs into asphalt mixture significantly improved the indirect tensile strength: 1.0 wt% of GnPs increased the indirect tensile strength of unmodified asphalt mixture from 0.79 to 1.1 MPa recording ~40% increment. 

The results of this study can confirm that graphene platelets have positive effect on the enhancement of the mechanical properties of asphalt mixture and its performance. However, further research is needed to quantify costs and savings of modifying asphalt by GnPs. Additionally, modifying the surface of graphene platelets with surfactants before the addition into bitumen is a potential research area; surface modification of GnPs can establish chemical bonding (covalent or ionic) with the matrix, thereby forming a strong interface between the two phases.

## Figures and Tables

**Table 1:** Aggregate properties.

Property	AASHTO Designation No.	Value
*Coarse aggregate*		
Specific Gravity	T-85	2.75
Water absorption %	T-85	3.5
Los Angeles Abrasion %	T-96	25
*Fine aggregate*		
Specific Gravity	T-84	2.67
Water absorption %	T-84	2.6

**Table 2:** Physical properties of unmodified asphalt.

Property	Value	Test Method	Specification Range
Penetration (@25 °C), 0.1 mm	67	ASTM D5	60–70
Softening point, °C	51	ASTM D36	45–55
Viscosity @135 °C, cP	422	ASTM D4402	–
Specific Gravity	1.03	ASTM D70	–
Flash point, °C	270	–	≥250

**Table 3:** TSRST results for modified and unmodified asphalt mixture.

Sample Composition	Fracture Cryogenic Stress, MPa	Fracture Temp., °C	Cryogenic Stress @ −20 °C, MPa
Unmodified asphalt mixture	4.3	−24.8	2.75
1.0 wt% GnPs-modified asphalt mixture	4.8	−26.9	2.5
